# Umbilical hernia with cholelithiasis and hiatal hernia: a clinical entity similar to Saint’s triad

**DOI:** 10.1186/s40792-015-0067-8

**Published:** 2015-08-12

**Authors:** Takahiro Yamanaka, Tatsuya Miyazaki, Yuji Kumakura, Hiroaki Honjo, Keigo Hara, Takehiko Yokobori, Makoto Sakai, Makoto Sohda, Hiroyuki Kuwano

**Affiliations:** Department of General Surgical Science (Surgery I), Gunma University Graduate School of Medicine, 3-39-22 Showa-machi, Maebashi, Gunma 371-8511 Japan

**Keywords:** Saint’s triad, Cholelithiasis, Hiatal hernia, Umbilical hernia

## Abstract

We experienced two cases involving the simultaneous presence of cholelithiasis, hiatal hernia, and umbilical hernia. Both patients were female and overweight (body mass index of 25.0–29.9 kg/m^2^) and had a history of pregnancy and surgical treatment of cholelithiasis. Additionally, both patients had two of the three conditions of Saint’s triad. Based on analysis of the pathogenesis of these two cases, we consider that these four diseases (Saint’s triad and umbilical hernia) are associated with one another. Obesity is a common risk factor for both umbilical hernia and Saint’s triad. Female sex, older age, and a history of pregnancy are common risk factors for umbilical hernia and two of the three conditions of Saint’s triad. Thus, umbilical hernia may readily develop with Saint’s triad. Knowledge of this coincidence is important in the clinical setting. The concomitant occurrence of Saint’s triad and umbilical hernia may be another clinical “tetralogy.”

## Background

Saint’s triad is characterized by the concomitant occurrence of cholelithiasis, hiatal hernia, and colonic diverticulosis. The etiology of Saint’s triad remains unclear. Saint’s triad has been extensively reported in Western countries, but there are few reports (only 20 cases) of Saint’s triad in Japan [1]. This condition has not been reported in Japan since it was described by Kabe et al. [2] in 1987. The number of patients with these three conditions is reportedly increasing, and detailed analysis indicates that Saint’s triad may not be a rare condition [2]. We suspect that many patients actually have Saint’s triad in Japan. However, Saint’s triad is less common than hiatal hernia, and colonic diverticulum has become a clinical problem because the ways to treatment of these diseases have been established. Therefore, the number of reports of Saint’s triad may be less than the number of actual cases.

We herein describe two patients with the simultaneous occurrence of cholelithiasis, hiatal hernia, and umbilical hernia. These patients had two of the three diseases of Saint’s triad (cholelithiasis and hiatal hernia). To the best of our knowledge, no previous reports have described the coexistence of umbilical hernia with any of the three conditions of Saint’s triad. We consider that the development of an umbilical hernia may be relevant to Saint’s triad. We herein discuss these two cases and present a brief review of the literature on Saint’s triad and umbilical hernia.

## Case presentation

### Case 1

A 79-year-old woman presented to another hospital for evaluation of abdominal pain after a meal. She had a history of hiatal hernia, gastroesophageal reflux disease, asthma, and pregnancy. She was taking a proton pump inhibitor by oral administration. She was a housewife and had no family history. Cholelithiasis and an umbilical hernia were detected by computed tomography (CT). She was referred to our hospital for surgical treatment of the hiatal hernia, umbilical hernia, and cholelithiasis. On physical examination, she was 141 cm tall, weighed 55 kg, and had a body mass index (BMI) of 27.7 kg/m^2^. Her abdomen was soft and slightly distended. The size of the umbilical hernia orifice was about 3 cm, and the skin around the umbilicus was protruded.

**Fig. 1:**
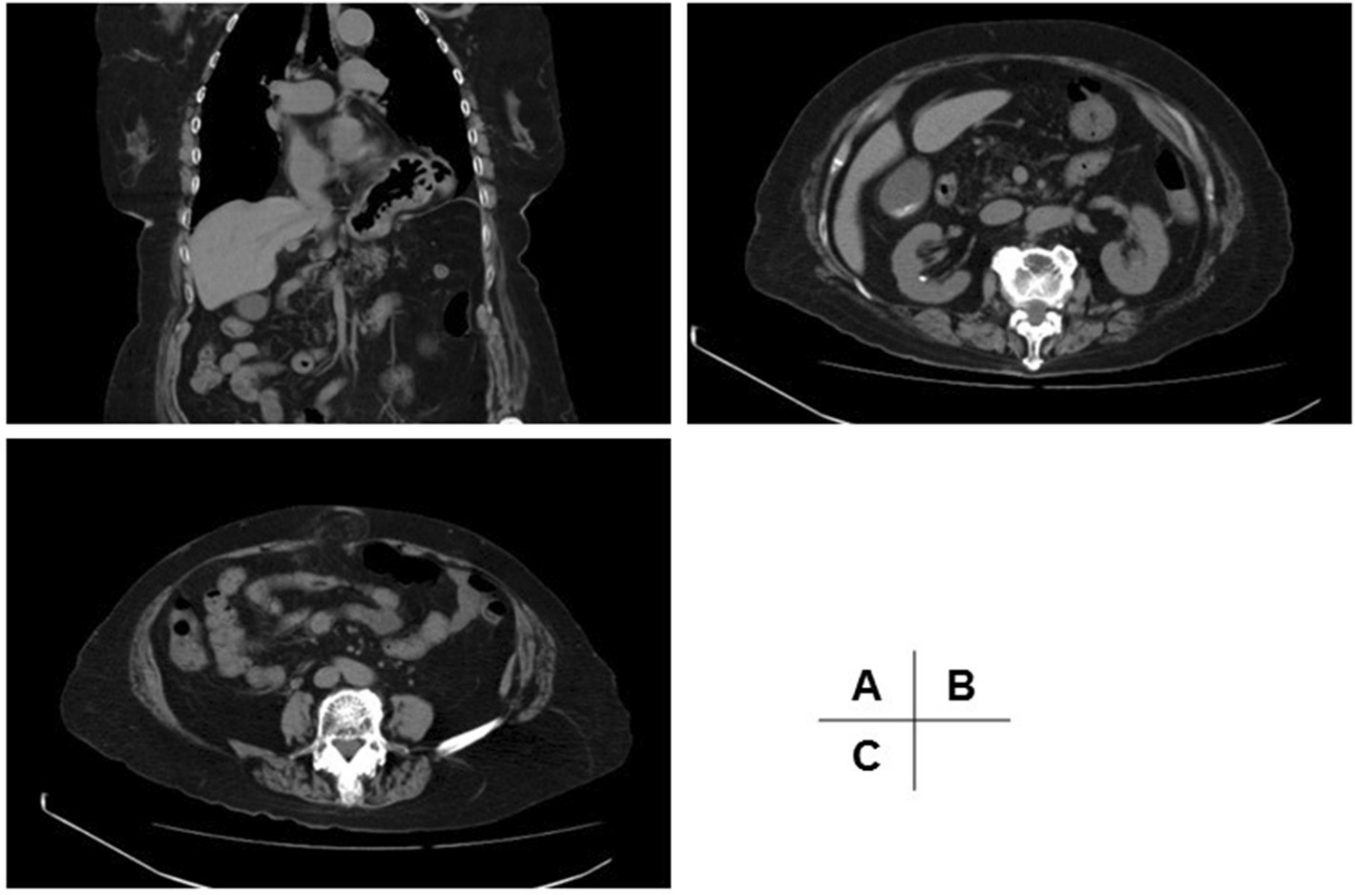
Images of Case 1. **a** CT revealed a hiatal hernia, through which the fundus of the stomach slid into the intrathoracic cavity. **b** CT revealed the presence of cholelithiasis. **c** Abdominal CT showed the umbilical hernia with accompanying prolapse of intestinal fat tissue

Laboratory test results were almost within normal limits. A chest radiograph revealed that the mediastinum was slightly extended by a hiatal hernia. Upper gastrointestinal endoscopy revealed a hiatal hernia of mixed type. The patient also had grade M reflux esophagitis according to the Los Angeles classification. Esophagography showed retention of contrast material in the intramediastinal stomach. However, no obstruction was present. CT confirmed the presence of the hiatal hernia (Fig. 1a), a stone within the gall bladder (Fig. 1b), and the umbilical hernia (Fig. 1c). The content of the umbilical hernia was intestinal fat. Colonoscopy revealed no diverticulosis. We performed laparoscopic hernioplasty of the hiatal hernia and Nissen fundoplication followed by laparoscopic cholecystectomy. Finally, we performed hernioplasty of the umbilical hernia and sutured the hernia orifice after resection of the sac. The postoperative course was uneventful, and the patient was discharged 13 days after the operation. She was still in good health 5 months after the operation.

### Case 2

A 41-year-old woman developed abdominal pain during treatment of a craniopharyngioma at a neurosurgery department. Cholecystitis and a gallstone were found by CT, and she underwent conservative therapy with antibacterial agents. Two months later, she desired surgical treatment for the cholelithiasis and was referred to our department. She had a history of pregnancy. On physical examination, she was 157 cm tall, weighed 72.4 kg, and had a BMI of 29.4 kg/m^2^. Her abdomen was soft and flat, and no obvious umbilical hernia was present.

**Fig. 2:**
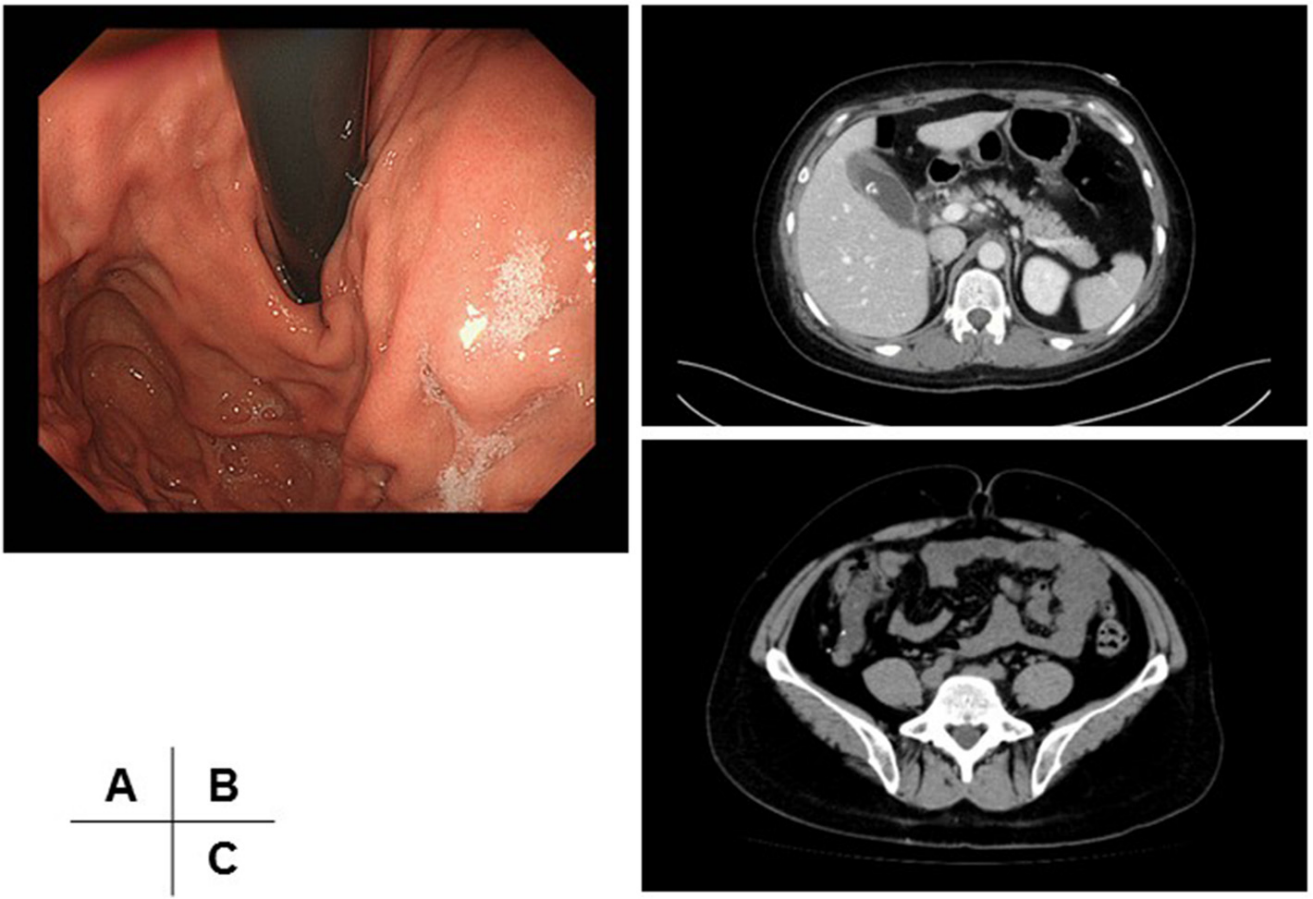
Images of Case 2. **a** Upper gastrointestinal endoscopy showed a slight hiatal hernia. **b** CT revealed cholelithiasis. **c** Abdominal CT identified a small umbilical hernia

Laboratory test results were almost within normal limits. A chest radiograph revealed elevation of the diaphragm. Upper gastrointestinal endoscopy revealed a sliding hiatal hernia (Fig. 2a). There was no reflex esophagitis. CT showed a stone within the gall bladder (Fig. 2b) and a small umbilical hernia (Fig. 2c) with an orifice size of 1 cm. Intestinal fat was present within the umbilical hernia. CT revealed no obvious diverticulosis. We performed laparoscopic cholecystectomy followed by hernioplasty of the umbilical hernia, and the hernia orifice was sutured after resection of the sac. The patient had no symptoms of gastroesophageal reflux disease or features of reflux esophagitis; therefore, we decided that she did not need to undergo laparoscopic hernioplasty for the hiatal hernia. The postoperative course was uneventful, and the patient was discharged 4 days after the operation. She was still in good health 4 months after the operation.

## Conclusions

Charles F.M. Saint was the first Chairman of Surgery at Cape Town University, who first noticed the concomitant occurrence of cholelithiasis, hiatal hernia, and colonic diverticulosis in the 1940s [3]. Muller, who was one of his students, described the phenomenon he termed Saint’s triad in 1948. Foster and Knutson [4] investigated the frequency of occurrence of these three conditions among many patients and found a 16.4, 14.0, and 18.7 % incidence of cholelithiasis, hiatal hernia, and colonic diverticulosis, respectively. They estimated that the incidence of these three diseases occurring simultaneously but unrelated with one another was 0.4 %. However, they found that these conditions occurred simultaneously and in association with one another at an incidence of 3.4 %. Muller [3] suspected that abdominal stress, such as that associated with constipation or delivery, was a causative factor. Palmer [5] reported that there might be some underlying etiologic factor common to all three conditions. However, Hilliard et al. [6] reported that there is no pathophysiological basis for the coexistence of Saint’s triad and cited Occam’s razor: “Plurality must not be posited without necessity.” In the most recent report on the etiology of Saint’s triad, Hauer-Jensen et al. [7] stated that “herniosis, the systemic connective tissue disease known to cause colonic diverticulosis and hernia, may be responsible for Saint’s triad.”

We experienced two cases involving patients with conditions similar to Saint’s triad. These two patients had common backgrounds; both were female, overweight (BMI of 25.0–29.9 kg/m^2^), and had a history of pregnancy and surgical treatment of cholelithiasis. Therefore, we investigated the patients and the risk factors for each of their diseases (Table 1).

**Table 1:** Risk factors for Saint’s triad and umbilical hernia in both patients

	Hiatus hernia	Cholelithiasis	Diverticulosis	Umbilical hernia	Case 1	Case 2
Age		aged	aged	aged	79	41
Sex		female	female	female	female	female
Obesity (BMI)	+	+	+	+	27.7	29.4
Pregnancy	+	+		+	+	+
Race		Pime Indians and Native American	Western		Japan	Japan
Constipation			motility			
etc.	taruma, congenital malformation	physical activity	diet, physical activity, western lifestyle	ascites abdominal distension		

The main risk factors for cholelithiasis are age [8], female sex [9], genetic factors (Pima Indians and certain other Native Americans are at higher risk) [10], pregnancy [11], obesity [12], and physical activity [13]. The main risk factors for colonic diverticulosis are age [14], female sex, age of >50 years [15], diet [16], obesity [17], colonic motility [18], Western lifestyle [19], physical activity [20], and smoking [21]. The risk factors for hiatal hernia, however, are speculative; a few reports have identified obesity [22] and pregnancy [23] as risk factors. The risk factors for umbilical hernia in adults are age [24], female sex [25], obesity [26], abdominal distension, ascites, and pregnancy [25]. To the best of our knowledge, there are no reports on the coexistence of umbilical hernia and any of these three diseases.

Although there is no obvious evidence about the risk factors for hiatal hernia, obesity is thought to be the only risk factor in common with those of Saint’s triad (cholelithiasis, hiatal hernia, and colonic diverticulosis). Pregnancy, female sex, and age are risk factors for three of the four conditions (Saint’s triad and umbilical hernia). Most people diagnosed with Saint’s triad are reportedly women aged >60 years [1, 2, 4]. Therefore, when a patient has these risk factors, some of the four above-mentioned diseases may appear simultaneously.

Both of the patients reported herein underwent surgical treatment for cholelithiasis. Patients with Saint’s triad reportedly require surgery for cholelithiasis; hiatal hernia and colonic diverticulosis are usually treated conservatively [1, 2]. Saint’s triad may be identified during a general preoperative examination. CT should be performed with consideration of these four diseases, and additional examinations may be needed (e.g., upper gastrointestinal endoscopy, colonoscopy, or colonography). We do not believe that surgery is always necessary for patients with no symptoms of either cholelithiasis or umbilical hernia. However, if one disease has an indication for treatment, we may be able to simultaneously treat another disease. A mild umbilical hernia can be easily repaired during the performance of hernioplasty and cholecystectomy, as in the present cases. In Japan, it is expected that the number of patients with these conditions will increase because of the growing prevalence of a Western lifestyle and the aging of society. Therefore, knowledge of these risk factors will help to diagnose the patient’s pathological condition.

In summary, we experienced two cases in which cholelithiasis, hiatal hernia, and umbilical hernia occurred simultaneously. It is important to remember that these four diseases (Saint’s triad and umbilical hernia) may readily appear simultaneously. The concomitant occurrence of these four diseases may be another clinical “tetralogy.”

## Consent

Written informed consent was obtained from the patients for publication of this case report and any accompanying images. A copy of the written consent is available for review by the Editor-in-Chief of this journal.

## Abbreviations

BMI: body mass index
CT: computed tomography
